# The influence of porosity on nanoparticle formation in hierarchical aluminophosphates

**DOI:** 10.3762/bjnano.10.191

**Published:** 2019-09-25

**Authors:** Matthew E Potter, Lauren N Riley, Alice E Oakley, Panashe M Mhembere, June Callison, Robert Raja

**Affiliations:** 1 Department of Chemistry, University of Southampton, Highfield Campus, Highfield, Southampton, SO17 1BJ, UK; 2 UK Catalysis Hub, Research Complex at Harwell, Rutherford Appleton Labs, Harwell Campus, OX11 0FA, UK; 3 Department of Chemistry, University College London, 20 Gordon Street, London, WC1H 0AJ, UK

**Keywords:** aluminophosphate, catalysis, hierarchical catalysts, nanoparticles, porosity

## Abstract

In this work we explore the deposition of gold onto a silicoaluminophosphate, using a variety of known nanoparticle deposition techniques. By comparing the gold particles deposited on a traditional microporous aluminophosphate, with an analogous hierarchical species, containing both micropores and mesopores, we explore the influence of this dual porosity on nanoparticle deposition. We show that the presence of mesopores has limited influence on the nanoparticle properties, but allows the system to maintain porosity after nanoparticle deposition. This will aid diffusion of reagents through the system, allowing continued access to the active sites in hierarchical systems, which offers significant potential in catalytic oxidation/reduction reactions.

## Findings

The controlled synthetic design of metallic nanoparticles has generated significant interest in recent decades due to their implementation in a range of fields, including medicine [1], optics [2] and catalysis [3]. Given the wide range of controllable properties, such as size, shape and charge, nanoparticle design is non-trivial, as specific procedures are constantly being developed to promote targeted features and behaviours [4]. Commonly, in catalysis nanoparticles are immobilised onto a solid support, preventing aggregation, leading to increased catalyst lifetime and performance [5]. Yet, immobilisation further complicates nanoparticle design by introducing surface–nanoparticle interactions, which have been shown to have a significant influence on the catalytic efficacy [6]. Commonly, the supports used are porous, which allows the nanoparticle to be deposited, and yet sufficiently isolated from other particles, to hinder aggregation. Of the wide range of supports utilised in the literature, micro- and meso-porous species are the most common [7–10]. Microporous materials can achieve high levels of control in catalytic reactions, resulting in targeted product selectivity and hence are, in principle, excellent hosts for metal nanoparticles [11]. Similarly, zeotype materials such as zeolites and aluminophosphates (AlPOs) also possess a wide range of secondary functionalities that could synergise with the nanoparticles in a catalytic reaction [12]. However, due to the limited pore aperture and channels of these systems, even sub-nanometre particles can block the framework and hinder activity, thereby preventing reagents from accessing the internal active sites. In contrast, mesoporous species (pores greater than 2 nm) maintain a large portion of their porosity when hosting metal nanoparticles, although they lack the more subtle ability to control the space around the active site [13]. In our previous work we have shown that inclusion of a micellular agent, i.e., dimethyloctadecyl[3-(trimethoxysilyl)propyl]ammonium chloride (DMOD) in an AlPO synthesis, alongside a microporous template, allows silanol-lined mesopores to form simultaneously with the microporous network, yielding a hierarchically porous (HP) system [14]. In this work, we utilise both a HP silicoaluminophosphate (SAPO) system and a traditional microporous SAPO-5 species, to demonstrate the advantages of hierarchical systems for nanoparticle deposition. We selected SAPO-5 as our basic framework, as the AlPO-5 synthesis is robust and forms one of the largest AlPO frameworks (pore size 7.3 Å), which will aid nanoparticle deposition and maintain porosity. Specifically, we compare three known methods for nanoparticle preparation, incipient wetness (IW), wet impregnation (WI) and ammonia evaporation (AE), on the typical microporous (MP-SAPO-5) and corresponding hierarchically porous system (HP-SAPO-5) [15–16].

**Figure 1:**
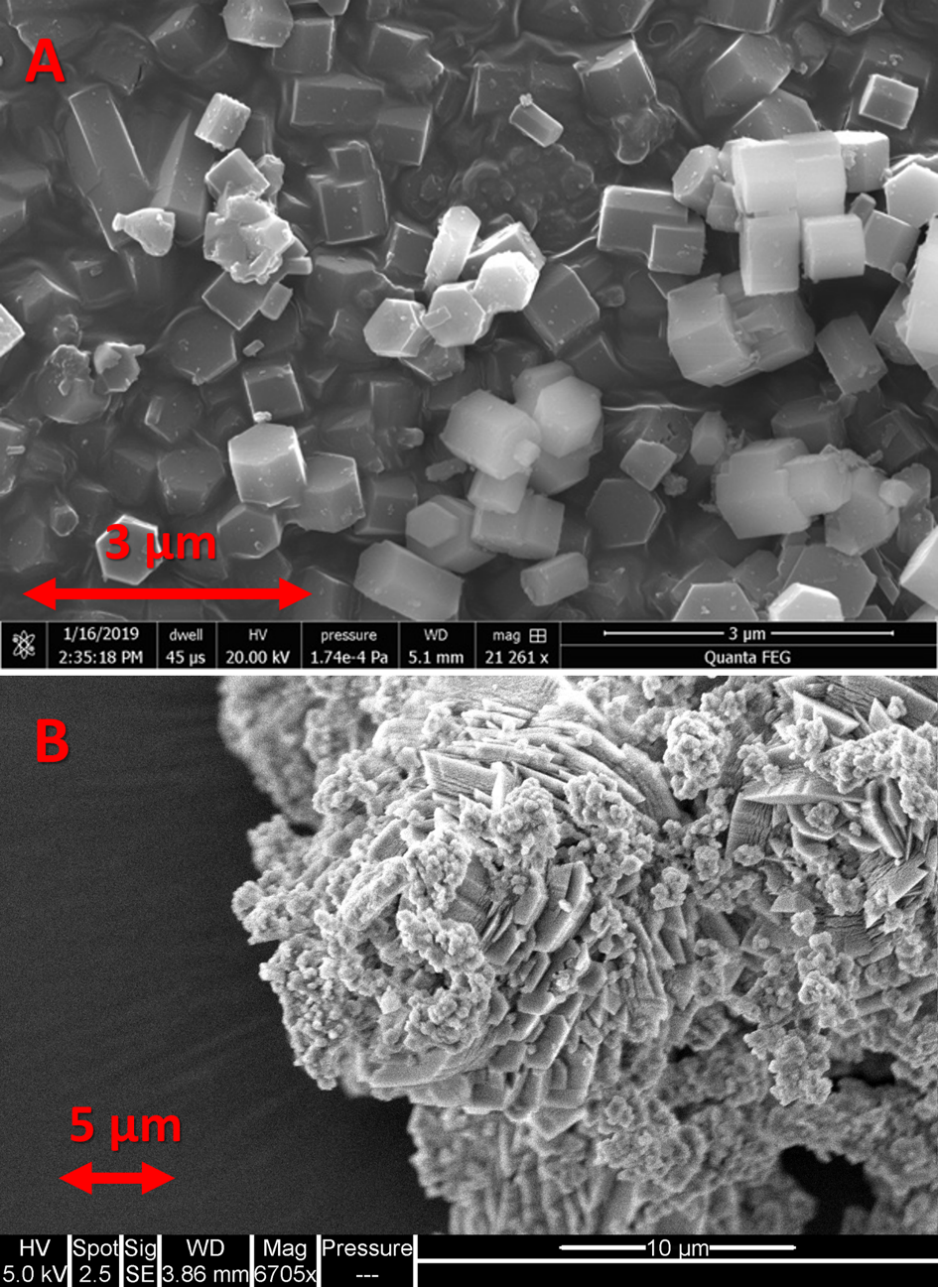
SEM images of microporous MP-SAPO-5 (A) and hierarchical HP-SAPO-5 (B).

MP-SAPO-5 was synthesised according to our previous work [17], giving the expected phase-pure, crystalline, microporous AlPO-5 framework (Figure 1A and Figures S1–S3 and Table S2, Supporting Information File 1). Modifying the synthesis procedure by adding a small quantity of DMOD (Table S1, Supporting Information File 1) into the hydrothermal gel (HP-SAPO-5) preserved the phase purity, as only AlPO-5 features are present (Figure S1, Supporting Information File 1). Nitrogen physisorption measurements show that while the type-I isotherm of MP-SAPO-5 strongly indicates microporosity, HP-SAPO-5 has a type-IV isotherm, indicating the hierarchical nature of the sample (Figure S2, Supporting Information File 1), but with a disordered mesoporous region. This is typical of hierarchical materials prepared in this manner [12,14] as they contain a broad range of mesopore sizes. Subsequent pore-distribution plots show no textural features for MP-SAPO-5 in the region of 20–350 Å, but HP-SAPO-5 shows a discerning hysteresis, indicating mesopores which are 60 Å in size (Figure S3, Supporting Information File 1). This is also highlighted as both systems possess similar micropore pore volumes, though HP-SAPO-5 has a much larger mesopore pore volume. The particles of HP-SAPO-5 were notably less crystalline than MP-SAPO-5 (Figure 1). HP-SAPO-5 showed crystalline features with smaller crystalline particles aggregating, forming part of a platelet morphology. The features of HP-SAPO-5 are attributed to the DMOD used in the synthesis. DMOD is believed to modify the crystallisation rate, which allows mesopores to form. In doing so, it also increases the disorder in the system, leading to agglomeration and different crystalline phases. Overall, we concluded that bare HP-SAPO-5 and MP-SAPO-5 systems were successfully synthesised. They were then used as supports for gold deposition in order to explore the influence that the microporous and hierarchical systems had on the nanoparticles characteristics.

All three deposition methods (IW, WI and AE) were carried out on both HP-SAPO-5 and MP-SAPO-5 with an intended theoretical loading of 1 wt % Au. Metal analysis on the deposited MP-SAPO-5 (Table S3, Supporting Information File 1) shows that the gold loadings vary significantly depending on the immobilisation strategy used, with IW being the most effective (0.66 wt % Au) and WI being the least (0.10 wt % Au). Likely the minimal amount of solvent used in the IW method increases support–metal interactions leading to more rapid deposition. The minimal amount of solvent will also be readily adsorbed into the internal pores of the material by capillary action, encouraging the metal to deposit on the micropores and mesopores and not just on the external surface. In contrast, the excess solvent used in WI will promote deposition primarily on the external surface. AE results in a reasonable deposition efficiency (0.49 wt % Au). Likely the evaporation stage of this process also encourages limited capillary action, similar to IW. Excellent agreement is seen between analogous MP-SAPO-5 and HP-SAPO-5 systems (Table S3, Supporting Information File 1), suggesting that the inclusion of silanol-lined mesopores neither encourages nor hinders nanoparticle deposition.

**Figure 2:**
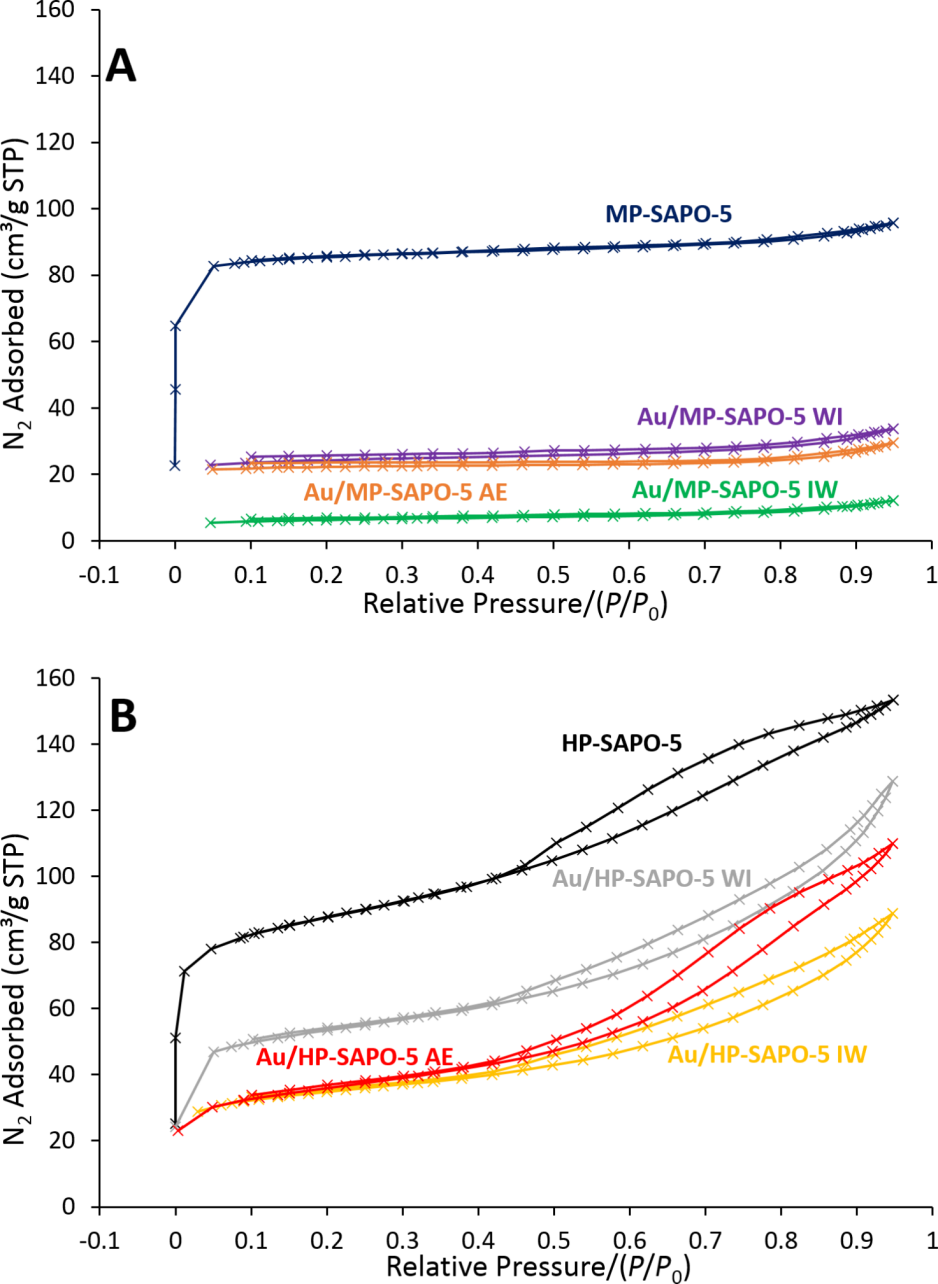
Nitrogen physisorption isotherms of gold-deposited microporous (A) and hierarchical (B) SAPO-5 systems showing that porosity is maintained in hierarchical HP-SAPO-5, but not in microporous MP-SAPO-5.

Following nanoparticle deposition, all samples maintained a phase-pure AlPO-5 framework, with the powder XRD patterns showing no significant variation in crystallinity or signal width (Figure 2 and Figure S4 and Figure S5, Supporting Information File 1). Nanoparticle deposition was found to greatly reduce the porosity of both the hierarchical and microporous supports. For MP-SAPO-5 the surface area decreases from 254 m^2^/g to just 72 m^2^/g, on depositing 0.10 wt % of Au through WI (Figure S6 and Table S4, Supporting Information File 1). This is accompanied by a significant decrease in pore volume (Table S4, Supporting Information File 1). Given that the framework integrity is maintained (Figure S4 and Figure S5, Supporting Information File 1), the decrease in porosity suggests that the 1D channels are blocked, restricting access to the internal micropores. As pore mouths are known to produce high-energy defect sites [18], they are more likely to encourage nanoparticle deposition, thus blocking the AlPO-5 channels. The IW and AE methods decrease the surface area to a greater extent due to increased Au deposition. The surface area follows a similar trend for HP-SAPO-5, with bare support > WI > AE > IW, again in agreement with the Au loadings (Figure 2B, Tables S3 and Table S4, Supporting Information File 1). Notably, a higher proportion of porosity is maintained in the hierarchical systems, where equivalent loadings of gold give surface areas above 110 m^2^/g. The surface areas for the Au-doped MP systems were difficult to estimate due to a loss of data at the very lowest pressures. However, the measurements here provide a reasonable estimate (Table S4, Supporting Information File 1). Further qualitative comparison of the isotherms for the Au-doped MP and HP at low pressure confirms the significantly lower surface area in the MP systems. The total pore volume follows a similar trend, with higher Au loadings prompting lower pore volumes. However, for the hierarchical system a higher proportion of the pore volume still remains on deposition (Table S4, Supporting Information File 1). Thus the introduction of mesopores into the hierarchical system (Figure S7, Supporting Information File 1) helps the systems maintain higher pore volumes and surface areas after nanoparticle inclusion. In principle this should translate into the hierarchical systems being improved catalysts with better diffusion.

**Figure 3:**
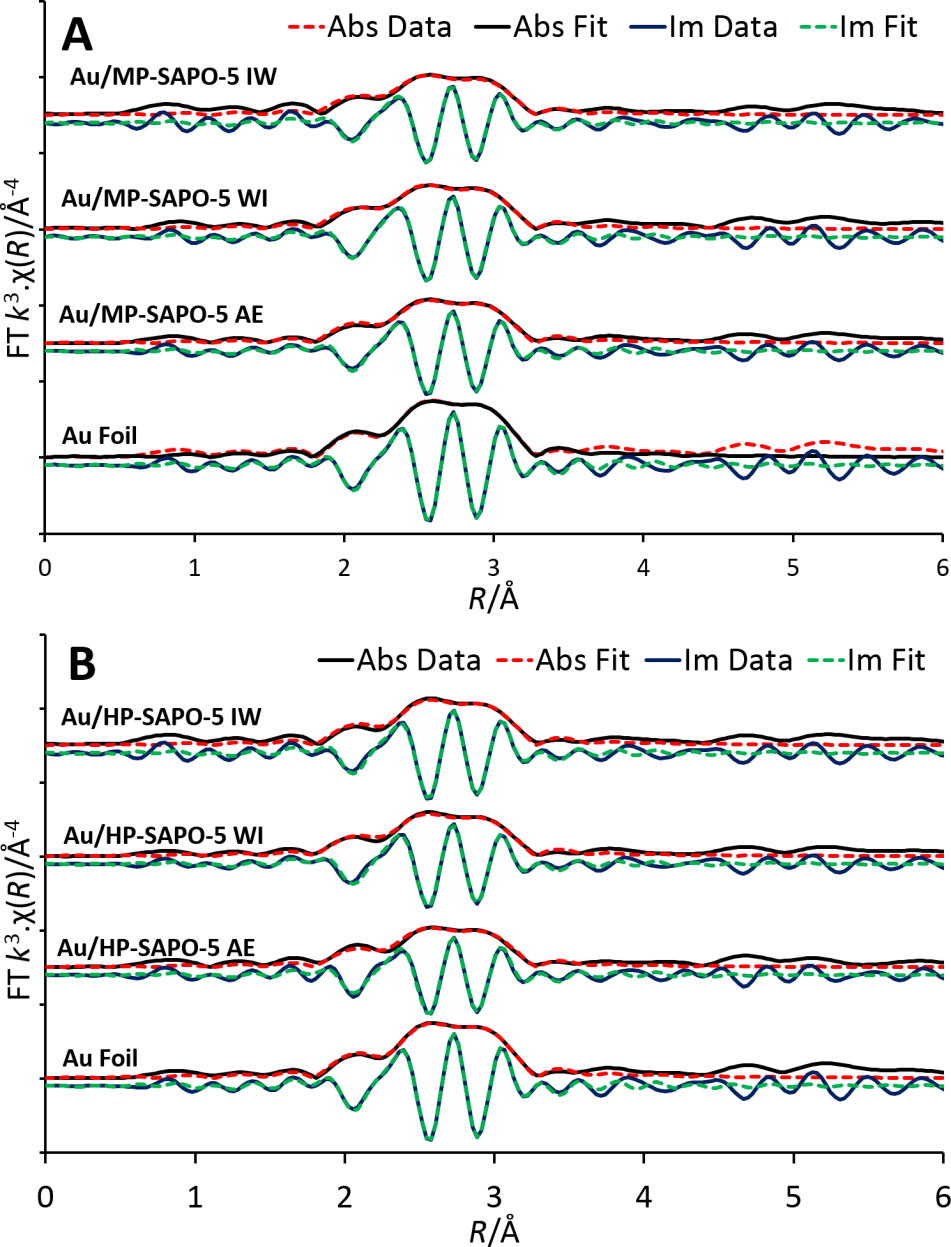
The magnitude and imaginary component of the k^3^-weighted Fourier transform for the XAS data of the Au-deposited microporous MP-SAPO-5 (A) and hierarchical HP-SAPO-5 (B) compared to the Au foil. Associated scattering paths, with a single Au–Au feature are included.

To probe the influence of the support on the deposited metal, a range of characterisation techniques were used to explore the nature of the Au species. UV–vis measurements show signals attributed to localised surface plasmon resonance for both Au/MP-SAPO-5 (Figure S8, Supporting Information File 1) and Au/HP-SAPO-5 (Figure S9, Supporting Information File 1) systems suggesting that nanoparticles have indeed formed via the IW and AE procedures [19]. However, no signals are seen for WI samples due to the low (0.10 wt %) Au loadings. The peak positions are in good agreement for the Au/MP-SAPO-5 species between the two techniques (IW 514 nm, AE 517 nm), though the hierarchical system shows a greater disparity (IW 511 nm, AE 529 nm). This is likely due to the wider range of possible deposition sites and environments, though overall the systems are in good agreement. X-ray adsorption spectroscopy (XAS) was used to probe the gold species, but only subtle variations between the systems was observed (Figure 3 and Figures S10–S15, Supporting Information File 1). There was good agreement with the Au foil, suggesting the gold has been successfully reduced to metallic gold particles. The Au/MP-SAPO-5 systems show a lower-energy X-ray absorption near edge structure (XANES), suggesting a higher average oxidation state (Au^δ+^, Figure S11, Supporting Information File 1) than the Au foil (Au^0^) [20]. In all cases the data was satisfactorily fit with a single Au–Au path, at a bond distance of 2.85–2.86 Å (Figure 3 and Table 1). The Au coordination number for all Au/SAPO-5 systems was found to be lower than the theoretical value of 12 for bulk Au foil, suggesting the formation of non-bulk Au systems. In both systems the coordination number was found to vary as AE < IW < WI, indicating AE produces smaller sized nanoparticles [21], despite WI having significantly lower Au loading. This again emphasises the influence of synthesis protocols on active site design.

**Table 1:** XAS fitting paths of Au-doped SAPO systems and Au foil.^a^

sample	abs–sc	*N*	*R*/Å	2σ^2^/Å^2^	*R*_factor_

Au/MP-SAPO-5 IW	Au–Au	10.0 (5)	2.849 (5)	0.0088 (5)	0.019
Au/MP-SAPO-5 WI	Au–Au	11.2 (3)	2.850 (3)	0.0089 (4)	0.006
Au/MP-SAPO-5 AE	Au–Au	10.1 (3)	2.852 (3)	0.0083 (3)	0.007

Au/HP-SAPO-5 IW	Au–Au	10.7 (4)	2.855 (5)	0.0082 (4)	0.015
Au/HP-SAPO-5 WI	Au–Au	10.8 (4)	2.851 (4)	0.0087 (5)	0.012
Au/HP-SAPO-5 AE	Au–Au	9.3 (4)	2.854 (5)	0.0082 (3)	0.020

Au foil	Au–Au	12 (fixed)	2.857 (2)	0.0077 (1)	0.004

^a^Fitting parameters: S_0_^2^ value of 0.826, determined by Au foil standard; fit range 3.0 < *k* < 12.3 and 1.5 < *R* < 3.5, number of independent points = 11.7, abs–sc = absorbing atom–scattering atom.

**Figure 4:**
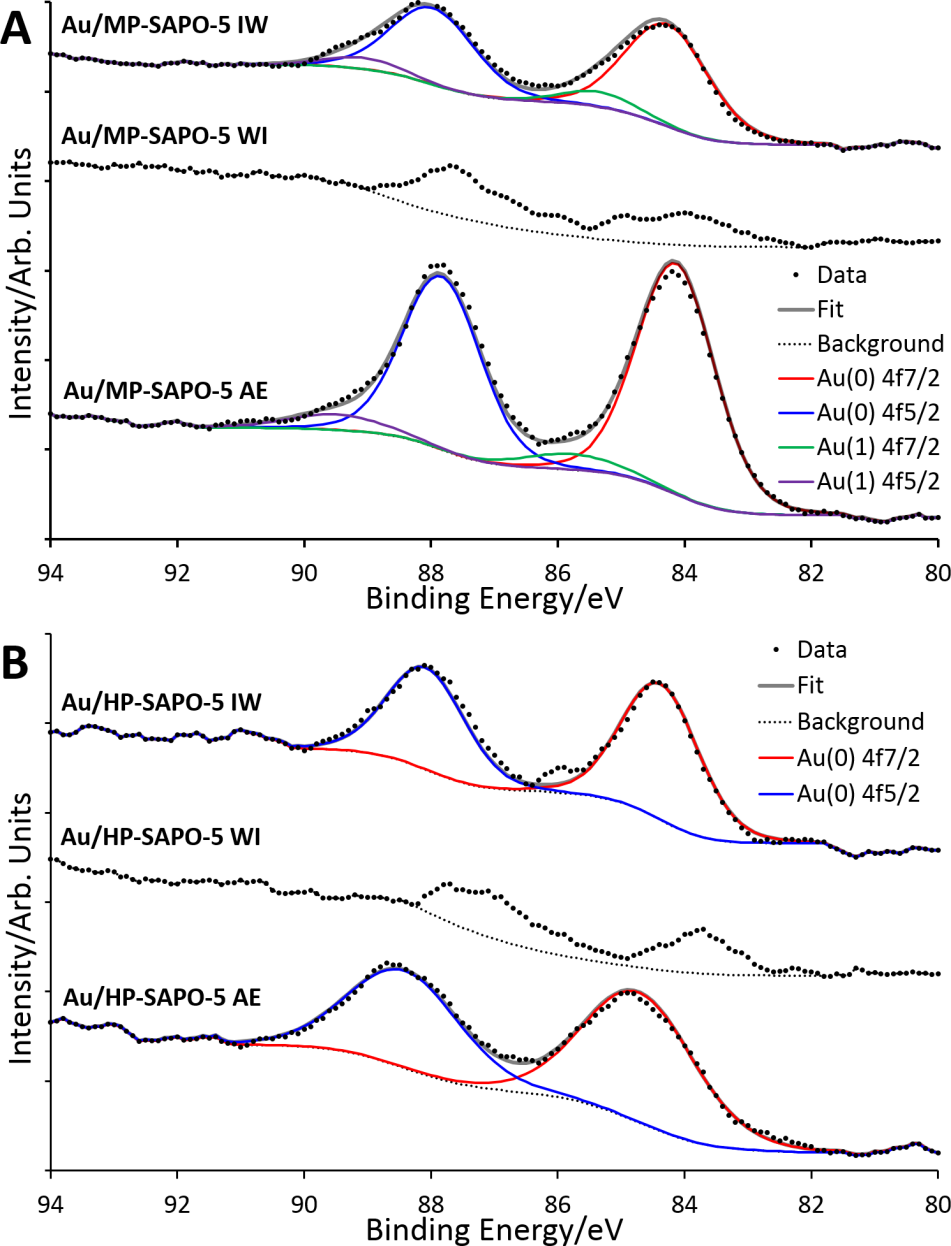
Stacked XPS data for Au-doped microporous MP-SAPO-5 (A) and hierarchical HP-SAPO-5 (B) showing the oxidation states present in the samples.

X-ray photoelectron spectroscopy (XPS) data (Figure 4) was in good agreement with the XAS data, as Au/HP-SAPO-5 IW and Au/HP-SAPO-5 AE systems were exclusively fit with Au^0^ features (Figure 4B). However, the corresponding microporous systems required additional Au^1^ features to be accurately fit, in agreement with the Au^δ+^ species observed from XANES (Figure 4A). As XPS has a limited penetration depth, it will accentuate surface species, which are more likely to contain Au^1^ species, over the bulk [22]. In both cases the weak signal from the low loading of the WI systems makes fitting infeasible. These measurements confirm that the different porosity of the two systems has only a subtle influence on the nanoparticle environment and coordination. A potential cause of this being the lack of porosity in the Au/MP-SAPO-5 species, hindering the complete reduction of the Au species, during the activation (calcination/reduction) process.

We have thus shown that pore blockage can be minimised by immobilising metal nanoparticles onto hierarchical systems, allowing tailored zeotype catalysts to act as hosts through the inclusion of mesopores, with their inherent porosity aiding nanoparticle reduction. Such materials have potential in catalytic oxidations/reductions, with Au/HP-SAPO-5 IW yielding a turn over number (TON) of 35 (Table S5, Supporting Information File 1) for the catalytic oxidation of toluene (preliminary findings). These materials offer significant potential as catalysts in their own right for C–H activation, but also as nanoparticle hosts. The dual porosity opens up the possibility of selectively isolating distinct active sites in specific-sized pores, towards intelligently designed bifunctional and tandem catalysts.

## Supporting Information

File 1 Additional experimental data.
