# Supplementary material for: The influence of porosity on nanoparticle formation in hierarchical aluminophosphates
Source: Beilstein J Nanotechnol. 2019 Sep 25;10:1952–7. doi: 10.3762/bjnano.10.191 (PMC6774075; doi:10.3762/bjnano.10.191)
Supplement: File 1 — Additional experimental data. [file Beilstein_J_Nanotechnol-10-1952-s001.pdf]

BEILSTEIN JOURNAL OF NANOTECHNOLOGY

Supporting Information

for

The influence of porosity on nanoparticle formation in hierarchical aluminophosphates

Matthew E. Potter, Lauren N. Riley, Alice E. Oakley, Panashe M. Mhembere, June Callison and Robert Raja

Beilstein J. Nanotechnol. **2019,** *10,* 1952–1957. doi:10.3762/bjnano.10.191

Additional experimental data

License and Terms: This is a supporting information file under the terms of the Creative Commons Attribution License (http://creativecommons.org/licenses/by/4.0). Please note that the reuse, redistribution and reproduction in particular requires that the authors and source are credited.
The license is subject to the *Beilstein Journal of Nanotechnology* terms and conditions: (https://www.beilstein-journals.org/bjnano)

Contents

Title	Page no.
Experimental	S3
Comparison of bare MP-SAPO-5 and HP-SAPO-5 supports	S7
Physicochemical properties of Au/SAPO-5 systems	S9
Probing the gold species on deposited systems with UV–vis spectroscopy	S12
Probing the gold species on deposited systems with XAS spectroscopy	S13
Toluene oxidation	S16
References	S16

Experimental

SAPO-5 synthesis

As per our previous work [1], for the synthesis of MP-SAPO-5, pseudo boehmite (3.0 g), H_3PO_4 (5.8 g, 85 wt % in water) and deionised water (22.5 mL) were added to a small teflon beaker and left to stir for 4 h. Triethylamine (TEA) (5.0 g) and colloidal silica gel (1.5 g) were added dropwise sequentially to the gel and stirred for 2 h. The gel (Table S1) was then transferred into PTFE-lined autoclaves and heated at 200 °C for 24 h. For HP-SAPO-5, a similar experimental procedure was followed, except 42 wt % DMOD in methanol (3.4 mL) was added to the reaction mixture along with the TEA and colloidal silica gel (Table S1). Samples were then washed with 1 L of deionised water and dried at 70 °C overnight. They were then calcined at 600 °C in a flow of air at 2.5 °C/min for 16 h.

Table S1: Gel ratios for SAPO-5 catalysts.

system	gel composition
MP-SAPO-5	2 Al/2 P /0.4 Si/2 TEA/50 H_2O
HP-SAPO-5	2 Al/2 P/0.4 Si/2 TEA/50 H_2O/0.01 DMOD

Incipient wetness deposition (IW)

The support (0.5 g) was added to a round-bottom flask and stirred while 0.5 mL of a 0.05 M aqueous solution of $HAuCl_4 \cdot 3H_2O$, was added dropwise, equating to 1 wt % Au on the support. Deionised water was added dropwise until the support had been evenly wetted. This was left to stir at room temperature for 1 h, after which the temperature of the stirrer plate was set to 120 °C overnight. The solid was transferred onto a watch glass and left in the drying oven at 70 °C overnight. The material was then calcined at 300 °C in a flow of air at 2.5 °C/min for 16 h.

Wetness impregnation deposition (WI)

The support (0.5 g) was added to a round-bottom flask and stirred while 0.5 mL of a 0.05 M aqueous solution of $HAuCl_4 \cdot 3H_2O$, was added dropwise, equating to 1 wt % Au on the support. Ethanol (10 mL) was added dropwise and left to stir overnight. The solution was filtered and left to dry in an oven at 70 °C overnight. The material was then calcined at 300 °C in a flow of air at 2.5 °C/min for 16 h.

Ammonia evaporation (AE)

0.5 mL of a 0.05 M aqueous solution of $HAuCl_4.3H_2O$ was added dropwise, equating to 1 wt % Au on the support, to a round-bottom flask. Deionised water (10 mL) was added to the stirred gold solution, followed by three drops of 30 % NH_4OH solution to reach pH 11. The solution was left to stir for 30 min. The support (0.5 g) was added and the temperature was set to 70 °C. The pH value of the solution was tested every 30 min until neutralisation, at which point the solution was centrifuged with water three times and left to dry at 70 °C overnight. The material was then calcined at 300 °C in a flow of air at 2.5 °C/min for 16 h.

Powder XRD (XRD)

Powder XRD patterns were collected on a Bruker AXS D2 Phaser with Cu Kα radiation.

Nitrogen physisorption

A Micromeritics TriStar II 3020 surface area analyser was used for nitrogen physisorption at 77 K. Surfaces areas were calculated using the BET model [2] and the Barrett–Joyner–Halenda (BJH) method [3] was used to determine pore sizes and volumes.

Scanning electron microscopy (SEM)

SEM images were performed using a JSM-5900 LV SEM. The samples were sputtered with gold prior to the measurements.

Induced coupled plasma-mass spectrometry (ICP-MS)

10 mg of samples was first digested in 1 mL of concentrated HNO_3, 1 mL of concentrated HCl and 0.75 mL of concentrated HF. The samples were heated overnight at 120 °C to ensure complete digestion occurred. Samples were then diluted into 60 mL of deionised water and then diluted 1:100 into 3% HNO_3 in deionised water. These samples were then measured on a high-resolution ICP-MS Thermo ELEMENT 2XR, with appropriate standards for quantification. ICP analysis was performed at the National Oceanography Centre, Southampton with the kind help of Dr. Matthew Cooper.

UV–vis spectroscopy

UV/Vis spectra were obtained using a Perkin Elmer Lambda 35 spectrometer in diffuse reflectance mode, with appropriate background subtraction.

X-ray photoelectron spectroscopy (XPS)

XPS analysis was performed using a Thermo Scientific K-Alpha+ / NEXSA instrument equipped with a monochromated Al Kα (1486.6 eV) source at the EPSRC XPS User's Service (HarwellXPS), Research Complex at Harwell. A pass energy of 200 eV and a step size of 1.0 eV was employed for all survey spectra while a pass energy of 40 eV and a step size of 0.1 eV was used for high-resolution spectra of the elements of interest. XPS spectra were calibrated against the corresponding oxygen 1s peaks. The high-resolution spectra of the incipient wetness impregnated (IW) and ammonia evaporated (AE) samples were with the fitted with Shirley backgrounds before peak

fitting using CasaXPS software [4]. Suitable fits could not be generated using any of the standard backgrounds (Shirley, Tougaard, Linear) for the wetness impregnated (WI) samples.

X-ray absorption spectroscopy (XAS)

Au XAS studies were carried out on the B18 beamline at the Diamond Light Source, Didcot, UK. Measurements were performed using a QEXAFS set-up with a fast-scanning Si(111) or Si(311) double crystal monochromator. 100 mg of sample was pressed into a pellet 1.3 cm in diameter, split in half and stacked on top of each other to enhance the signal. Data was collected ex situ, in fluorescence mode using a 36 element Ge Monolithic detector, due to the low loading of gold in the sample. The Au L_3 edge was used, scanning from 11.719 to 12.700 keV, at a rate of 6 eV/second, at an increment of 0.5 eV. Au foil was used as the reference standard for aligning the energy of the samples. This was measured in transmission, simultaneously with the fluorescence sample measurements. On average, six scans were acquired to improve the signal-to-noise level of the data. XAS data processing and EXAFS analysis were performed using IFEFFIT with the Horae package (Athena and Artemis) [5,6]. The amplitude reduction factor, S_0^2, was derived from EXAFS data analysis of the Au foil, and used as a fixed input parameter. Beamtime was acquired on session SP19850-2, as part of our membership of the UK Catalysis Hub Beamtime Allocation Group.

Comparison of bare MP-SAPO-5 and HP-SAPO-5 supports

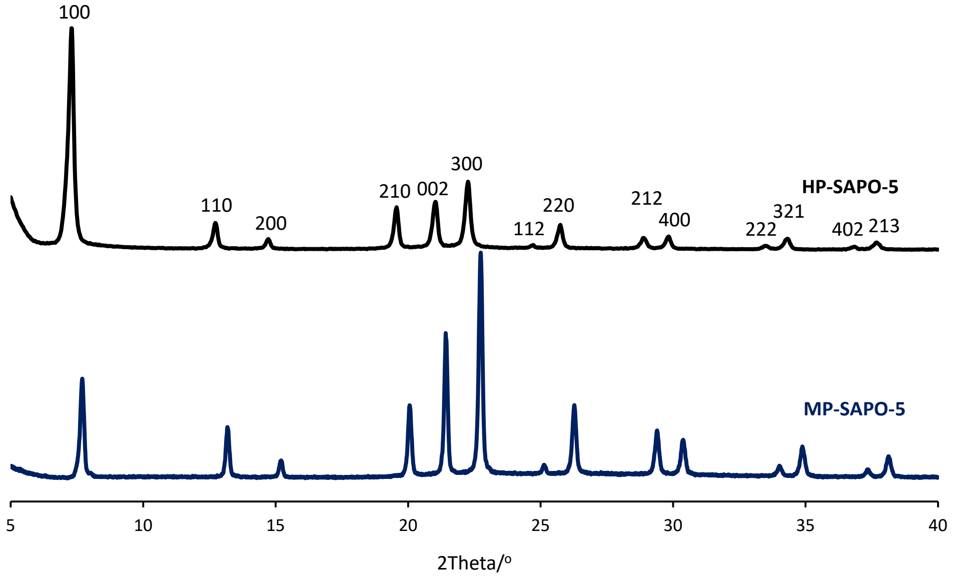

Figure S1: Powder XRD pattern comparing the microporous (MP-SAPO-5) and hierarchical (HP-SAPO-5) SAPO-5 systems.

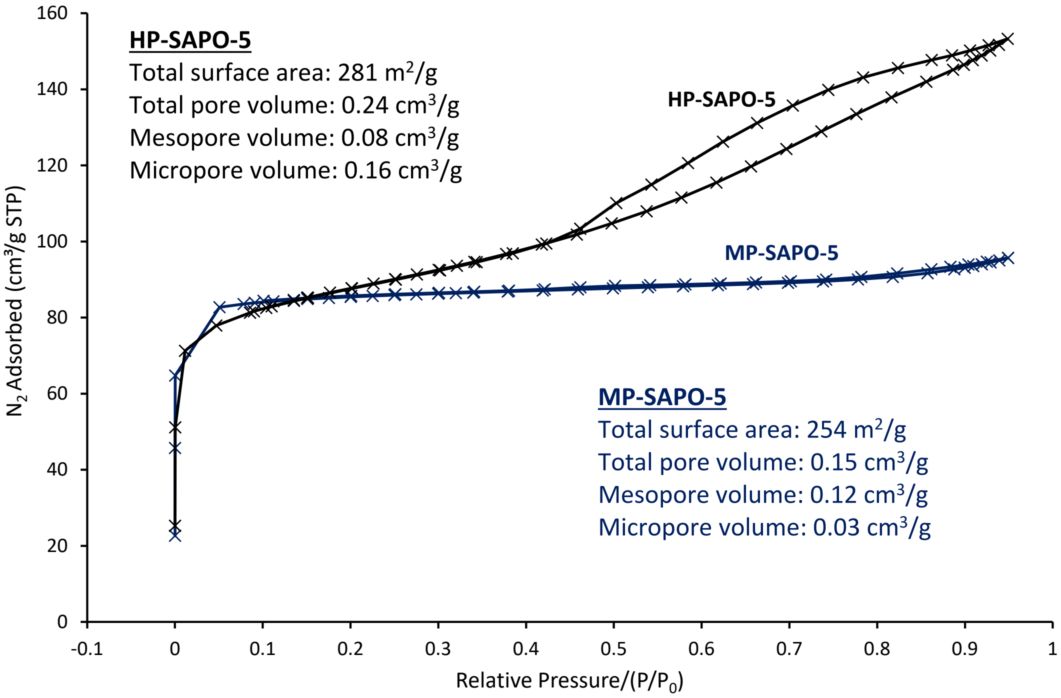

Figure S2: Nitrogen physisorption isotherms at 77 K comparing the microporous (MP-SAPO-5) and hierarchical (HP-SAPO-5) SAPO-5 systems.

Table S2: Contrasting the physicochemical properties of the microporous (MP-SAPO-5) and hierarchical (HP-SAPO-5) SAPO-5 systems.

	metal loading/wt %			physisorption data			
system	Al	P	Si	surface area $/m^2 \cdot g^{-1}$	total pore volume $/cm^3 \cdot g^{-1}$	micropore volume $/cm^3 \cdot g^{-1}$	mesopore volume $/cm^3 \cdot g^{-1}$
MP-SAPO-5	24.4	3.5	24.5	254	0.15	0.12	0.03
HP-SAPO-5	18.8	7.3	20.8	281	0.24	0.08	0.16

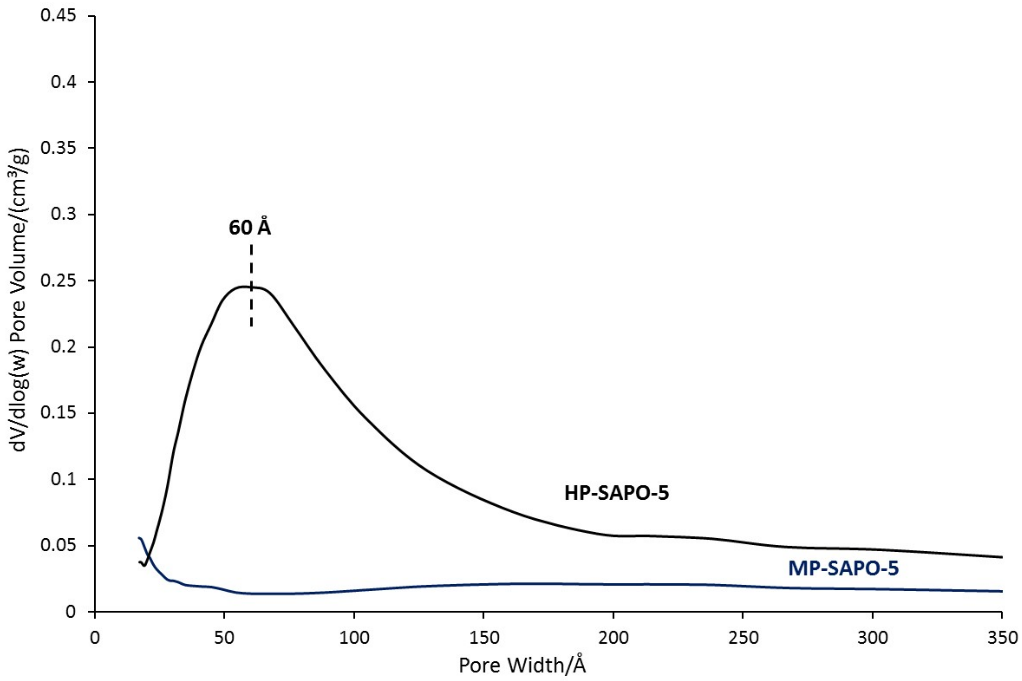

Figure S3: BJH pore-size distribution plot comparing the microporous (MP-SAPO-5) and hierarchical (HP-SAPO-5) SAPO-5 systems.

Physicochemical properties of Au/SAPO-5 systems

Table S3: Full ICP analysis of Au/SAPO-5 systems.

sample	Al/wt %	P/wt %	Si/wt %	Au/wt %
MP-SAPO-5	24.4	24.5	3.5	0.00
Au/MP-SAPO-5 AE	21.0	21.7	2.9	0.49
Au/MP-SAPO-5 WI	24.4	24.4	3.4	0.10
Au/MP-SAPO-5 IW	21.8	22.7	3.1	0.66
HP-SAPO-5	18.8	20.8	7.3	0.00
Au/HP-SAPO-5 AE	22.2	17.7	8.6	0.57
Au/HP-SAPO-5 WI	18.7	20.8	7.3	0.10
Au/HP-SAPO-5 IW	20.2	16.7	8.0	0.64

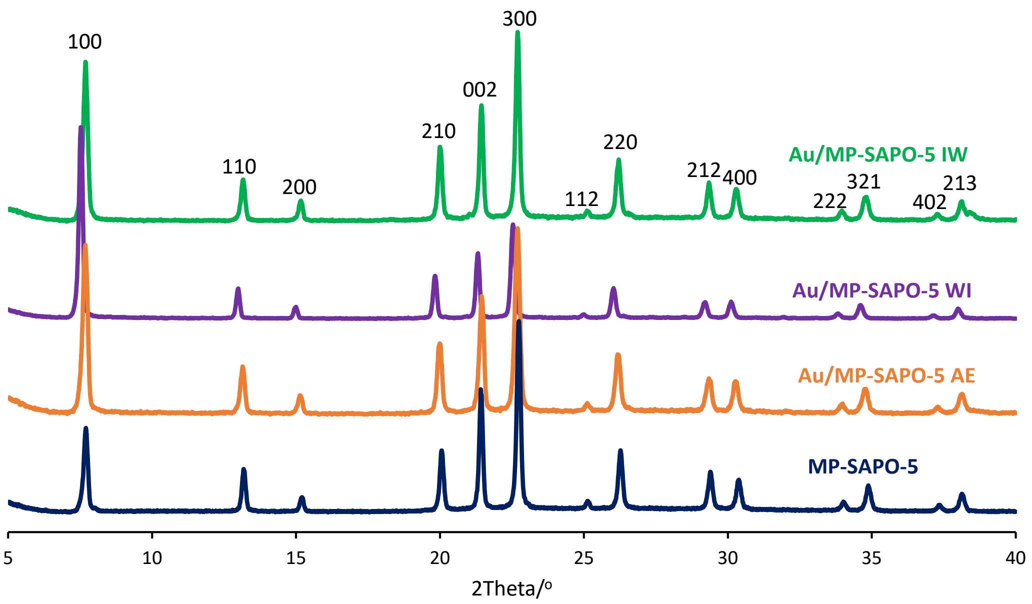

Figure S4: Powder XRD patterns showing the phase purity of MP-SAPO-5 is maintained on depositing Au onto the system.

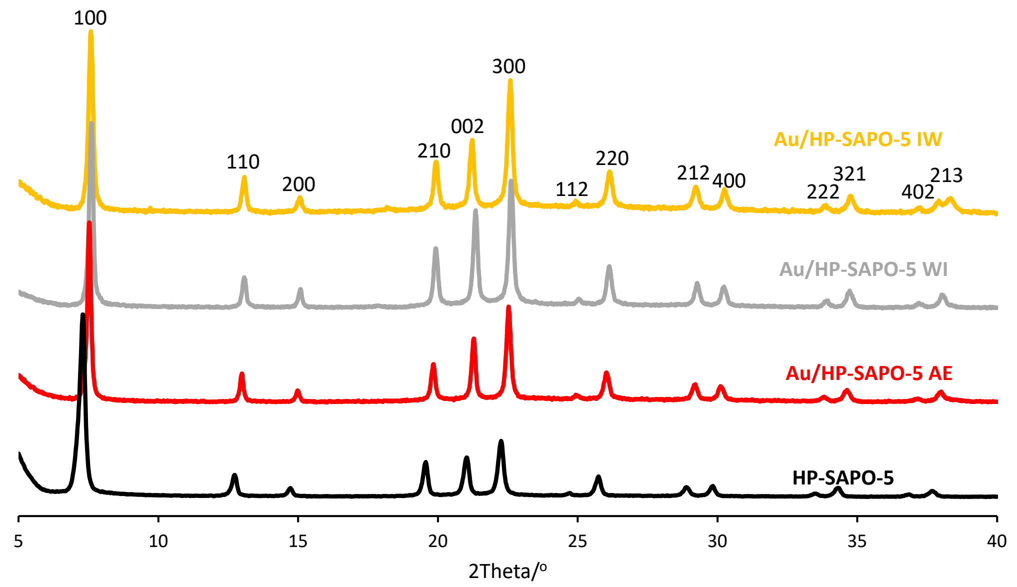

Figure S5: Powder XRD patterns showing the phase purity of HP-SAPO-5 is maintained on depositing Au onto the system.

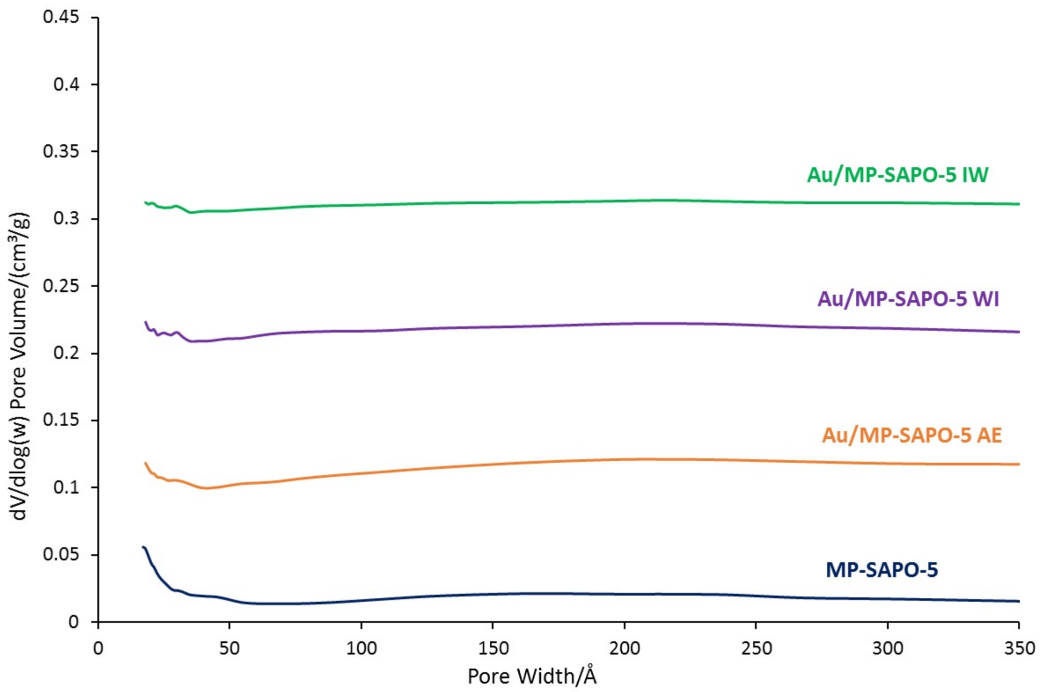

Figure S6: BJH pore size distribution functions of MP-SAPO-5 after Au deposition. Plots are stacked in increments of 0.05 cm^3/g for clarity.

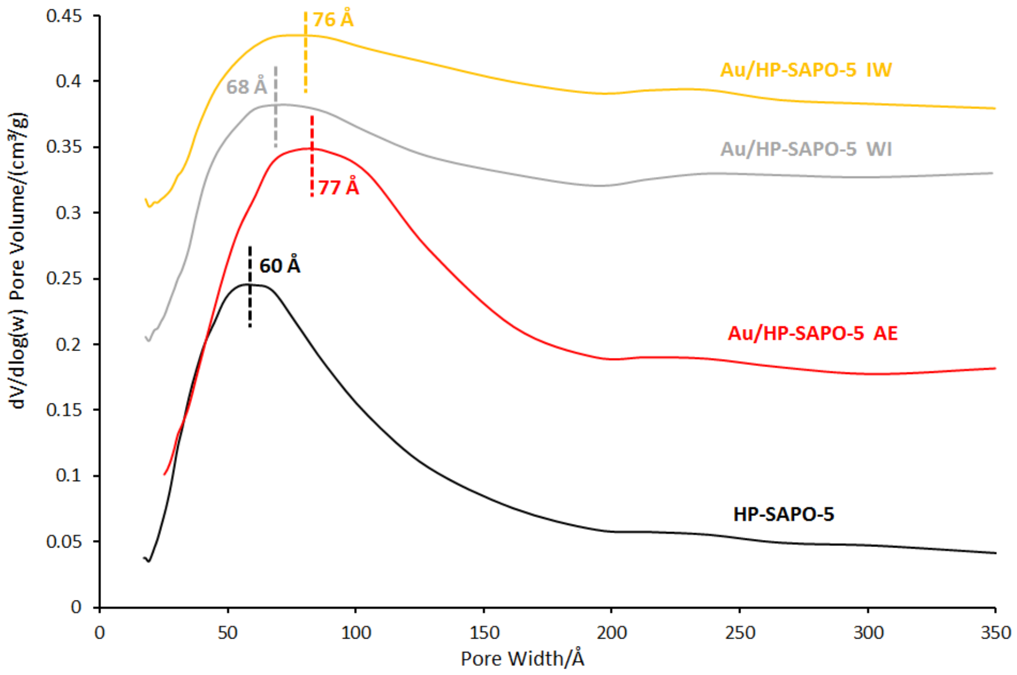

Figure S7: BJH pore size distribution functions of HP-SAPO-5 after Au deposition. Plots are stacked in increments of 0.05 cm^3/g for clarity.

Table S4: Comparing the nitrogen physisorption data of gold-doped SAPO-5 materials.

sample	surface area /$m^2 \cdot g^{-1}$	total pore volume /$cm^3 \cdot g^{-1}$	micropore volume /$cm^3 \cdot g^{-1}$	mesopore volume /$cm^3 \cdot g^{-1}$	pore width /Å
MP-SAPO-5	254	0.15	0.12	0.03	—
Au/MP-SAPO-5 AE	65	0.05	0.03	0.01	—
Au/MP-SAPO-5 WI	72	0.05	0.03	0.02	—
Au/MP-SAPO-5 IW	20	0.02	0.01	0.01	—
HP-SAPO-5	281	0.24	0.08	0.16	60
Au/HP-SAPO-5 AE	119	0.17	0.01	0.16	77
Au/HP-SAPO-5 WI	173	0.20	0.04	0.16	68
Au/HP-SAPO-5 IW	114	0.14	0.02	0.11	76

Probing the gold species on deposited systems with UV–vis spectroscopy

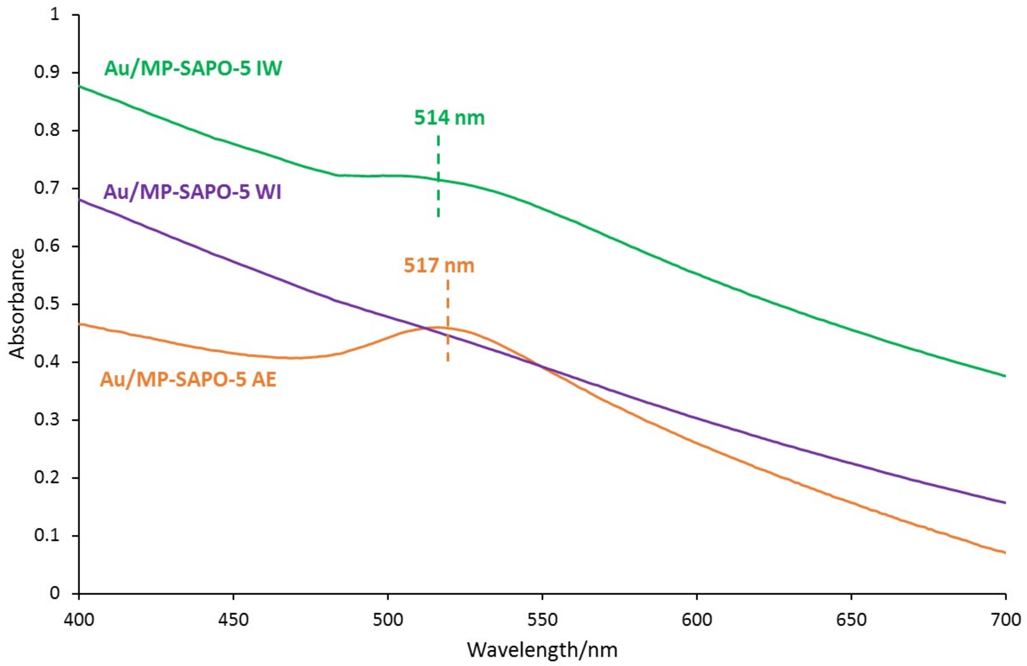

Figure S8: UV–vis spectra of Au deposited on microporous SAPO-5 systems. Plots are stacked for clarity.

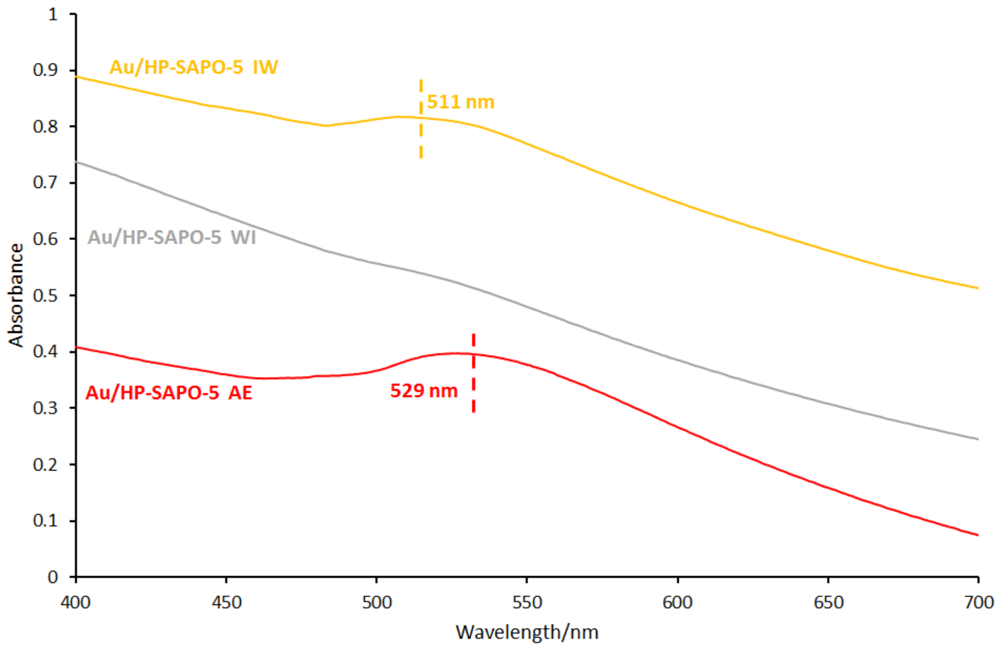

Figure S9: UV–vis spectra of Au deposited on hierarchical SAPO-5 systems. Plots are stacked for clarity.

Probing the gold species on deposited systems with XAS spectroscopy

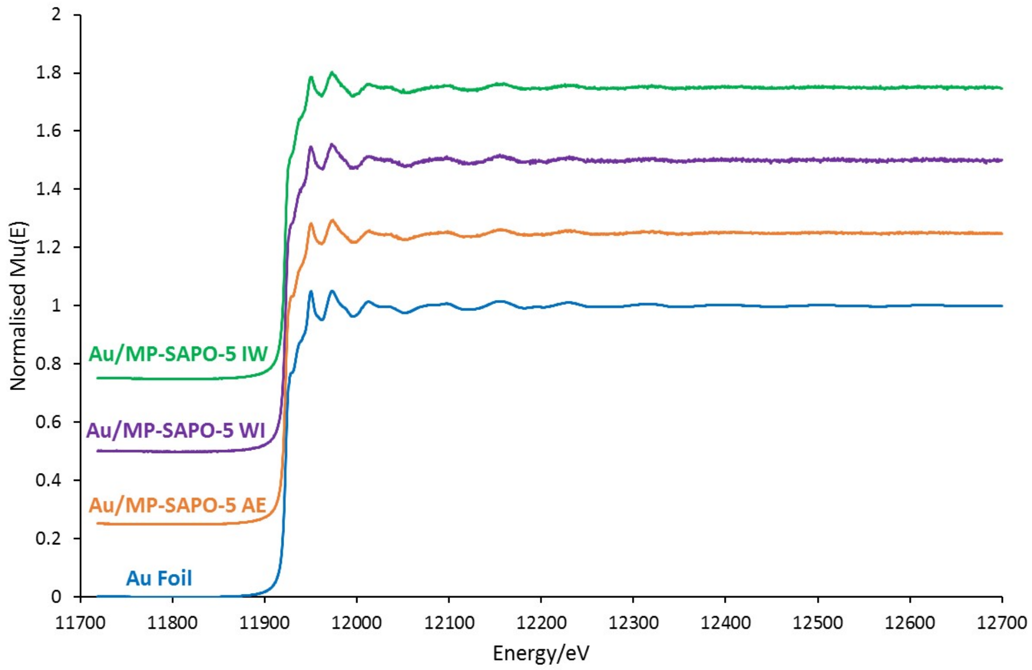

Figure S10: Normalised Mu(*E*) EXAFS plots in the Au L_3 region of Au deposited onto MP-SAPO-5, compared with the Au reference foil.

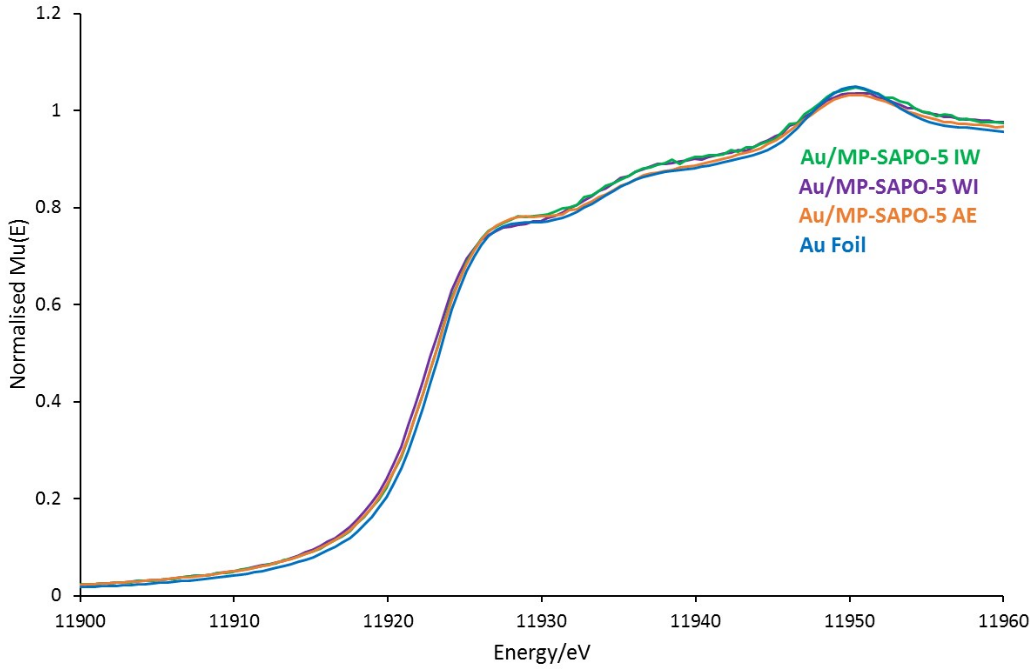

Figure S11: Normalised Mu(*E*) XANES plots in the Au L_3 region of Au deposited onto MP-SAPO-5, compared with the Au reference foil.

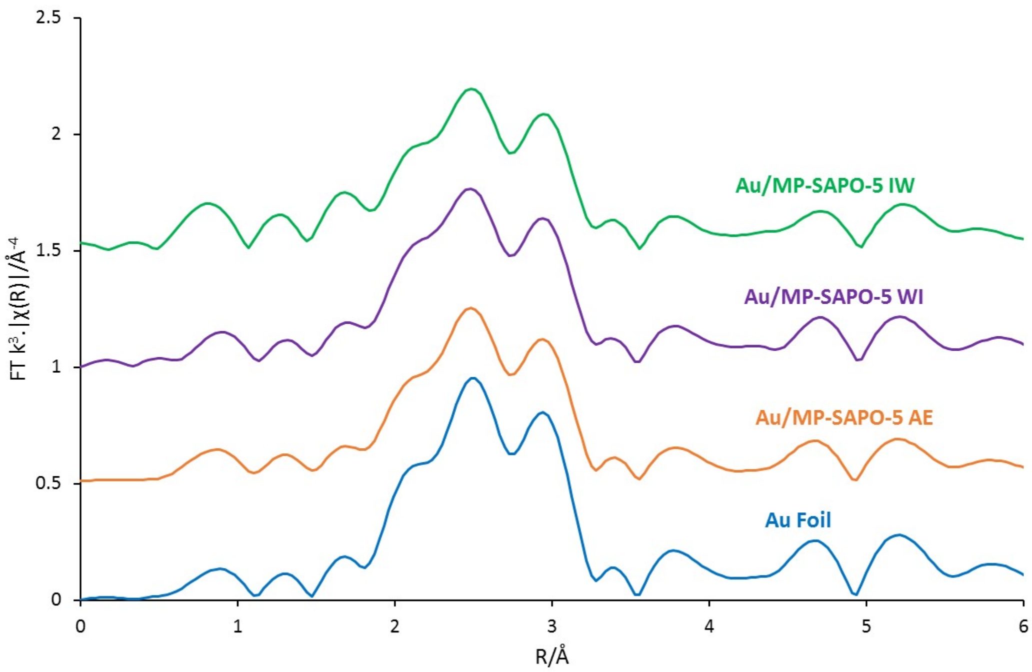

Figure S12: Magnitude of the k^3-weighted Fourier Transform for the EXAFS data on Au deposited onto MP-SAPO-5, compared with the Au reference foil.

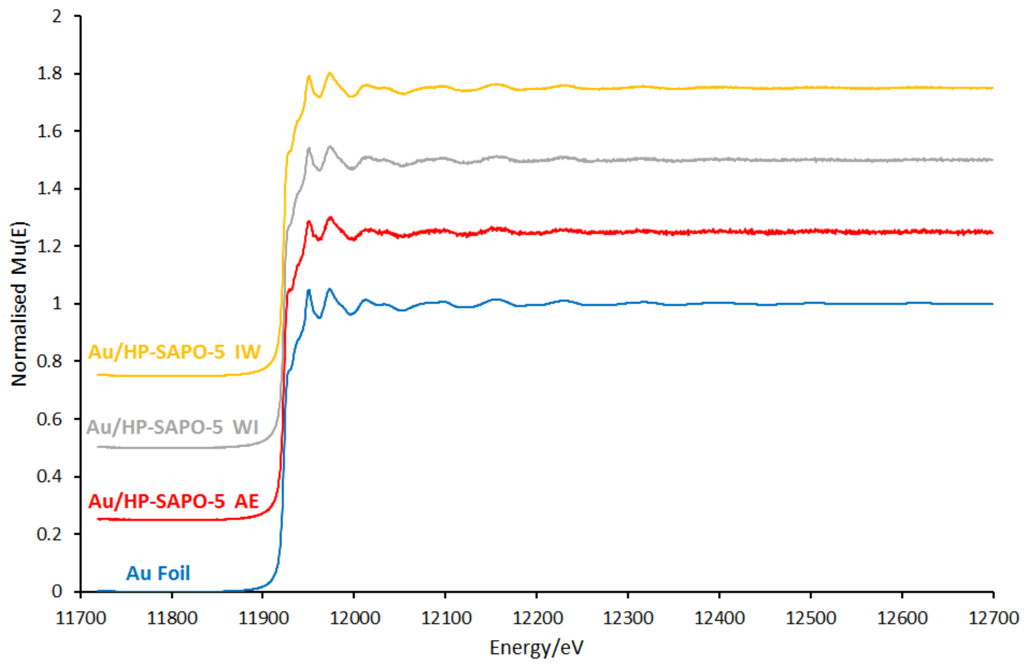

Figure S13: Normalised Mu(E) EXAFS plots in the Au L_3 region of Au deposited onto HP-SAPO-5, compared with the Au reference foil.

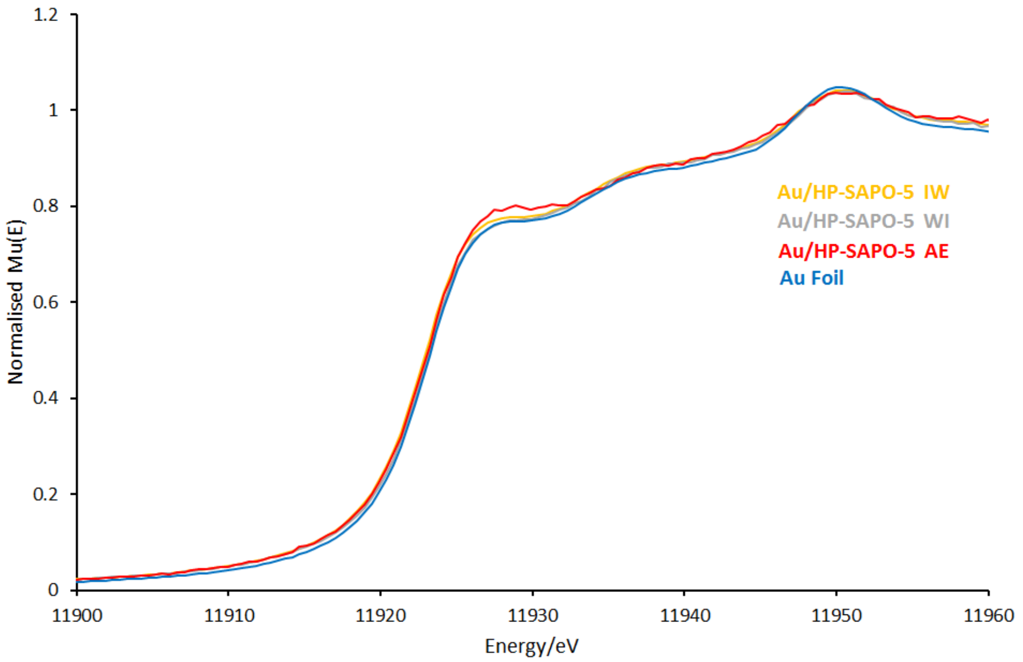

Figure S14: Normalised Mu(*E*) XANES plots in the Au L_3 region of Au deposited onto HP-SAPO-5, compared with the Au reference foil.

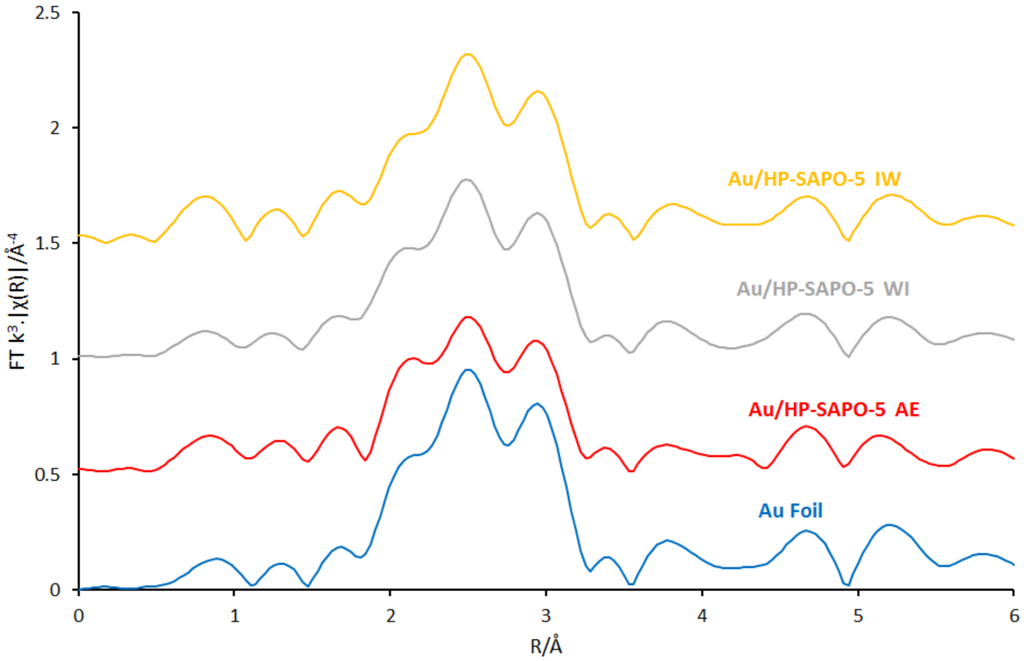

Figure S15: The magnitude of the k^3-weighted Fourier Transform for the EXAFS data on Au deposited onto HP-SAPO-5, compared with the Au reference foil.

Toluene oxidation

Toluene (16.5 mmol, 1.52 g), 70% TBHP in water (16.5 mmol, 2.28 mL) and bis(2-methoxyethyl) ether (diglyme) (1.9 mmol, 0.28 mL) were all added to a round-bottom flask (10 mL), which was then sealed and stirred at 80 °C in a pre-heated oil bath. 50 mg of catalyst was added to the mixture and left to stir for 24 h. The samples were analysed using a Perkin Elmer 3400CX gas chromatogram with flame ionisation detector (FID). Products were identified against authenticated standards, and quantified by via calibration of measured response factors against the diglyme internal standard.

Table S5: Preliminary toluene catalysis results.

Time/h	benzyl alcohol selectivity/mol %	benzoic acid/mol %	TON[a]
8	0	100	16
24	28	72	35

[a]Turnover number, moles of toluene converted/moles of gold

References

1. Potter, M. E.; Kezina, J.; Bounds, R.; Carravetta, M.; Mezza, T. M.; Raja, R. *Catal. Sci. Technol.* **2018,** *8,* 5155–5164. doi:10.1039/C8CY01370E
2. *CasaXPS,* version 2.3.12; Casa Software Ltd., 2000. http://www.casaxps.com
3. Brunauer, S.; Emmett, P. H.; Teller, E. *J. Am. Chem. Soc.* **1938,** *60,* 309–319. doi:10.1021/ja01269a023
4. Barrett, E. P.; Joyner, L. G.; Halenda, P. H. *J. Am. Chem. Soc.* **1951,** *73,* 373–380. doi:10.1021/ja01145a126
5. Newville, M. *J. Synchrotron Radiat.* **2001,** *8,* 322–324. doi:10.1107/S0909049500016964
6. Ravel, B.; Newville, M. *J. Synchrotron Radiat.* **2005,** *12,* 537–541. doi:10.1107/S0909049505012719
